# Solution-Processed Cu(In, Ga)(S, Se)_2_ Nanocrystal as Inorganic Hole-Transporting Material for Efficient and Stable Perovskite Solar Cells

**DOI:** 10.1186/s11671-017-1933-z

**Published:** 2017-02-28

**Authors:** Lu Xu, Lin-Long Deng, Jing Cao, Xin Wang, Wei-Yi Chen, Zhiyuan Jiang

**Affiliations:** 1 State Key Laboratory of Physical Chemistry of Solid Surfaces, Department of Chemistry, College of Chemistry and Chemical Engineering, Xiamen University, Xiamen, 361005 People’s Republic of China; 2 Pen-Tung Sah Institute of Micro-Nano Science and Technology, Xiamen University, Xiamen, 361005 People’s Republic of China

**Keywords:** Perovskite solar cells, Hole-transporting material, CIGSSe nanocrystals

## Abstract

Perovskite solar cells are emerging as one of the most promising candidates for solar energy harvesting. To date, most of the high-performance perovskite solar cells have exclusively employed organic hole-transporting materials (HTMs) such as 2,2′,7,7′-tetrakis-(*N*,*N*-di-*p*-methoxyphenylamine)-9,9′-spirobifluorene (spiro-OMeTAD) or polytriarylamine (PTAA) which are often expensive and have low hole mobility. Almost all these HTMs reported needed lithium salt, e.g., lithium bis(trifluoromethylsulfonyl)imide (Li-TFSI) doping, to improve hole mobility and performance. However, the use of Li-TFSI should be avoided because the hygroscopic nature of Li-TFSI could cause decomposition of perovskite and reduce device stability. Herein, we employed solution-processed CuIn_0.1_Ga_0.9_(S_0.9_Se_0.1_)_2_ (CIGSSe) nanocrystals as a novel inorganic HTM in perovskite solar cells. A power conversion efficiency of 9.15% was obtained for CIGSSe-based devices with improved stability, compared to devices using spiro-OMeTAD as HTM. This work offers a promising candidate of Cu-based inorganic HTM for efficient and stable perovskite solar cells.

**Electronic supplementary material:**

The online version of this article (doi:10.1186/s11671-017-1933-z) contains supplementary material, which is available to authorized users.

## Background

Nowadays, perovskite solar cells (PSCs) employing organo-lead halide perovskite absorber materials have attracted substantial attention because of their excellent qualities, such as large absorption coefficient [1, 2], direct bandgap [3], high charge carrier mobility [4], and long diffusion lengths [5, 6]. Methylammonium lead iodide (CH_3_NH_3_PbI_3_) is a light-harvesting perovskite material with a direct bandgap of 1.55 eV [7], which makes it an ideal light absorber in PSCs [8, 9]. Perovskite films can be prepared by using either one-step or two-step sequential deposition methods [10–12]. To boost the solar cell efficiency, various types of solar cell architectures including perovskite-sensitized solar cells, mesoporous PSCs, and planar n-i-p or p-i-n heterojunction solar cells have been developed [8, 9]. So far, the efficiencies of mesoporous PSCs are higher than those of the planar PSCs. Recent advances in perovskite materials, perovskite film deposition methods, and device structures have been proven to contribute to the dramatic progress in PSCs. Thus, the power conversion efficiency (PCE) of PSCs has been increased incredibly from 3.8% to over 20% in the past few years [1, 2, 13–19]. Noteworthy, organic small molecule 2,2′,7,7′-tetrakis-(*N*,*N*-di-*p*-methoxyphenylamine)-9,9′-spirobifluorene (spiro-OMeTAD) [2, 14–16] is the most widely used hole-transporting material (HTM) in these high-performance PSCs with mesoporous or planar n-i-p architectures. Other organic small molecules and polymers are also employed as HTMs in PSCs, such as poly(3-hexylthiophene) (P3HT) [20–22], polyaniline (PANI) [23], polytriarylamine (PTAA) [19, 20], and poly[2,1,3-benzothiadiazole-4,7-diyl-[4,4-bis(2-ethylhexyl)-4H-cyclopenta[2,1-b:3,4-b′]dithiophene-2,6-diyl]] (PCPDTBT) [20]. Despite the high efficiency obtained with these HTMs, the relatively high cost of these HTMs limits further large-scale production of PSCs because of their complicated synthetic procedures and high-purity requirement. Besides, almost all these HTMs require lithium salt, e.g., lithium bis(trifluoromethylsulfonyl)imide (Li-TFSI) doping, to improve hole mobility and performance [20]. However, the hygroscopic nature of Li-TFSI could cause decomposition of perovskite and reduce the device stability [24, 25]. Therefore, it is necessary to develop other HTMs with low cost and high stability.

Compared with organic HTMs, *p*-type inorganic HTMs have already been employed in PSCs due to their high stability and low cost. CuSCN [26], CuI [27, 28], PbS [29], and Cu-doped NiO [30] have been explored in PSCs with PCEs of 12.4, 6.0, 7.88, and 15.4%, respectively. Inspired from these previous reports about Cu-based inorganic HTMs in PSCs with high performance and good stability, in this study, we demonstrate that copper-based inorganic semiconductor CuIn_1 − *x*_Ga_*x*_(S_*y*_Se_1 − *y*_)_2_ (CIGSSe) nanocrystals can act as a new HTM in PSCs.

Cu-based chalcopyrite semiconductors such as CuInS_2_, Cu(In_*x*_Ga_1 − *x*_)Se_2_ (CIGS), and CIGSSe are promising light-absorbing materials due to their excellent optoelectronic properties, good photostability, and long-term stability [31, 32]. Recently, CuInS_2_ quantum dot was applied as a HTM in conventional PSCs to replace the organic hole conductor spiro-OMeTAD [33]. However, the relatively low PCE of CuInS_2_ suggests that there is still much room for further improvement in performance. Furthermore, the bandgap of CIGSSe can be tuned from 0.98 to 2.40 eV [34], which provides advantageous characteristics such as expanding the photoresponse of perovskite solar cells. Herein, we report the facile synthesis and application of solution-processed CIGSSe nanocrystals as a novel copper-based inorganic HTM in PSCs. Perovskite solar cells with mesoporous architecture were fabricated, and the CH_3_NH_3_PbI_3_ perovskites were formed by a one-step solvent-engineering technology [35]. A PCE of 9.15% was achieved by using CIGSSe nanocrystals as a HTM in PSCs with enhanced device stability under ambient conditions, compared to devices employing Li-TFSI-doped spiro-OMeTAD as HTM.

## Methods

### Synthesis of CIGSSe Nanocrystal

CIGSSe nanocrystals were synthesized using the methods reported in the literatures [34, 36] with some modification. Reactions were performed using a Schlenk manifold under a purified nitrogen atmosphere. In a typical synthesis, 1 mmol of CuCl (0.0990 g), 0.1 mmol of InCl_3_ (0.0221 g), 0.9 mmol of GaCl_3_ (0.159 g), 0.2 mmol of elemental Se (0.0158 g) and 1.62 mmol of elemental sulfur (0.0518 g), 0.09 mmol of 1,2-ethanedithiol (7.5 μL), and 25 mL oleylamine were added in a 50-mL three-necked flask. The flask was placed on a magnetic hot plate with continuous stirring and then attached to the Schlenk line. The temperature was monitored and controlled using a thermocouple and a PID temperature controller through the third neck of the flask. The flask was purged of oxygen and water by pulling vacuum for 1 h, followed by N_2_ bubbling at 130 °C for 1 h while stirring. Next, the system was slowly heated to 240 °C and kept for 4 h under vigorous stirring. After reaction, the flask was quenched in a cold water bath to quickly bring the temperature down. The CIGSSe nanocrystal/oleylamine mixture was transferred to a 50-mL centrifuge tube. Fifteen milliliters of ethanol was added to the mixture and centrifuged at 8000 rpm for 10 min. After such a washing step, the supernatant, containing unreacted precursor and byproducts, was discarded. The CIGSSe nanocrystals were in the precipitate. The nanocrystals were then redispersed in 10 mL of chloroform and centrifuged at 7000 rpm for 5 min to remove poorly capped nanocrystals and large particulates. The well-capped nanocrystals remained dispersed in the supernatant. The precipitate was discarded, and a small amount of oleylamine (0.2 mL) was subsequently added to the supernatant to ensure complete surface passivation of the nanocrystals. To remove excess capping ligands and remaining impurities, the product was again precipitated using ∼5 mL of ethanol and centrifuged at 8000 rpm for 10 min, then redispersed in chloroform. This process was done three times to obtain a high-purity product. The isolated nanocrystals were redispersed in toluene for further characterization.

### Preparation of Spiro-OMeTAD HTM

The spiro-OMeTAD solution was prepared by dissolving 72.3 mg of spiro-OMeTAD, 28.8 μL of 4-tert-butylpyridine, and 17.5 μL of lithium bis(trifluoromethylsulfonyl)imide (Li-TFSI) solution (520 mg Li-TFSI in 1 mL acetonitrile) in 1 mL of chlorobenzene. Spiro-OMeTAD HTM layer was deposited on top of the perovskite layer by spin-coating the mixture solution at 4000 rpm for 30 s.

### Device Fabrication

The etched fluorine-doped tin oxide (FTO) substrate was cleaned with detergent; then ultrasonicated in deionized water, acetone, and ethanol for 10 min every time; and subsequently dried by air. Finally, the substrate was treated with UV-ozone for 10 min. A thin layer (~100 nm) of compact TiO_2_ layer (bl-TiO_2_) was deposited onto FTO substrate by spin-coating of 0.15 M titanium diisopropoxide bis(acetylacetonate) in *n*-butyl alcohol at 3000 rpm for 30 s, followed by annealing at 125 °C for 10 min. This process was repeated for three times. Then, the film was annealed at 500 °C for 30 min. After cooling to room temperature, the film was immersed in 50 mM TiCl_4_ solution at 70 °C for 30 min and then washed with deionized water and ethanol. After drying, mesoporous TiO_2_ (mp-TiO_2_) film with thickness of ~200 nm was deposited on the compact layer by spin-coating TiO_2_ paste (Dyesol 18NR-T) diluted in ethanol at 1:5 by weight at 5000 rpm for 30 s. The layer was then sintered in air at 550 °C for 30 min. Four hundred sixty-one milligrams of PbI_2_, 159 mg of CH_3_NH_3_I, and 78 mg of DMSO (molar ratio 1:1:1) was mixed in 600 mg of *N*,*N*-dimethylformamide (DMF) solution at room temperature with stirring for 1 h in order to prepare a CH_3_NH_3_I•PbI_2_•DMSO adduct solution. The completely dissolved solution was spin-coated on the mesoporous TiO_2_ layer at 4000 rpm for 25 s, and 0.5 mL of diethyl ether was slowly dripped on the rotating substrate in 10 s before the surface changed to be turbid caused by rapid vaporization of DMF. The transparent CH_3_NH_3_I•PbI_2_•DMSO adduct film was heated at 65 °C for 1 min and 100 °C for 2 min in order to obtain a dense CH_3_NH_3_PbI_3_ film. After the substrate was cooled down to room temperature, a CIGSSe dispersion (10 mg/mL in toluene) was deposited on top of the perovskite layer by spin-coating at 4000 rpm for 30 s, followed by annealing at 100 °C for 5 min. Reference devices based on spiro-OMeTAD HTM were also fabricated for comparison. Finally, an 80-nm gold layer was thermally evaporated on top of the device to form the back contact. The active area was 0.09 cm^2^.

### Measurements and Characterization

The current density-voltage (*J*-*V*) characteristics were recorded from a solar simulator equipped with a Keithley 2400 source meter and 300-W collimated xenon lamp (Newport) calibrated with the light intensity to 100 mW/cm^2^ at AM 1.5 G solar light condition by the certified silicon solar cell. The external quantum efficiency (EQE) measurement was measured using a Newport Oriel QE/IPCE measurement kit. The light intensity was calibrated using a single-crystal Si photovoltaic cell as the reference.

The surface morphology and cross-section images of the films were acquired using a SEM-4800 field-emission scanning electron microscope (HITACHI-S4800). Transmission electron microscopy (TEM) images were observed with transmission electron microscopy (JEM-1400). The X-ray diffraction (XRD) measurements were carried out by X-ray powder diffraction analysis (Rigaku, RINT-2500) with a Cu Kα radiation source. Ultraviolet-visible (UV-Vis) absorption spectra were measured on a Varian Cary 5000 UV-Vis-NIR spectrometer. The steady-state photoluminescence (PL) spectra were measured using an Edinburgh Instruments FLS920 spectrometer. The ultraviolet photoelectron spectroscopy (UPS) spectra were obtained by a Thermo Scientific ESCALAB 250Xi instrument using a HeI (21.2 eV) energy source. The electrochemical impedance spectra (EIS) were carried out under 100 mW/cm^2^ at AM 1.5 G simulated solar irradiation with a computer-controlled potentiostat (Metrohm Autolab PGSTAT204) at a bias potential of 0 V. AC 20-mV perturbation was applied with a frequency from 1 MHz to 10 Hz. The spectra were fitted using Z-View software.

## Results and Discussion

**Fig. 1:**
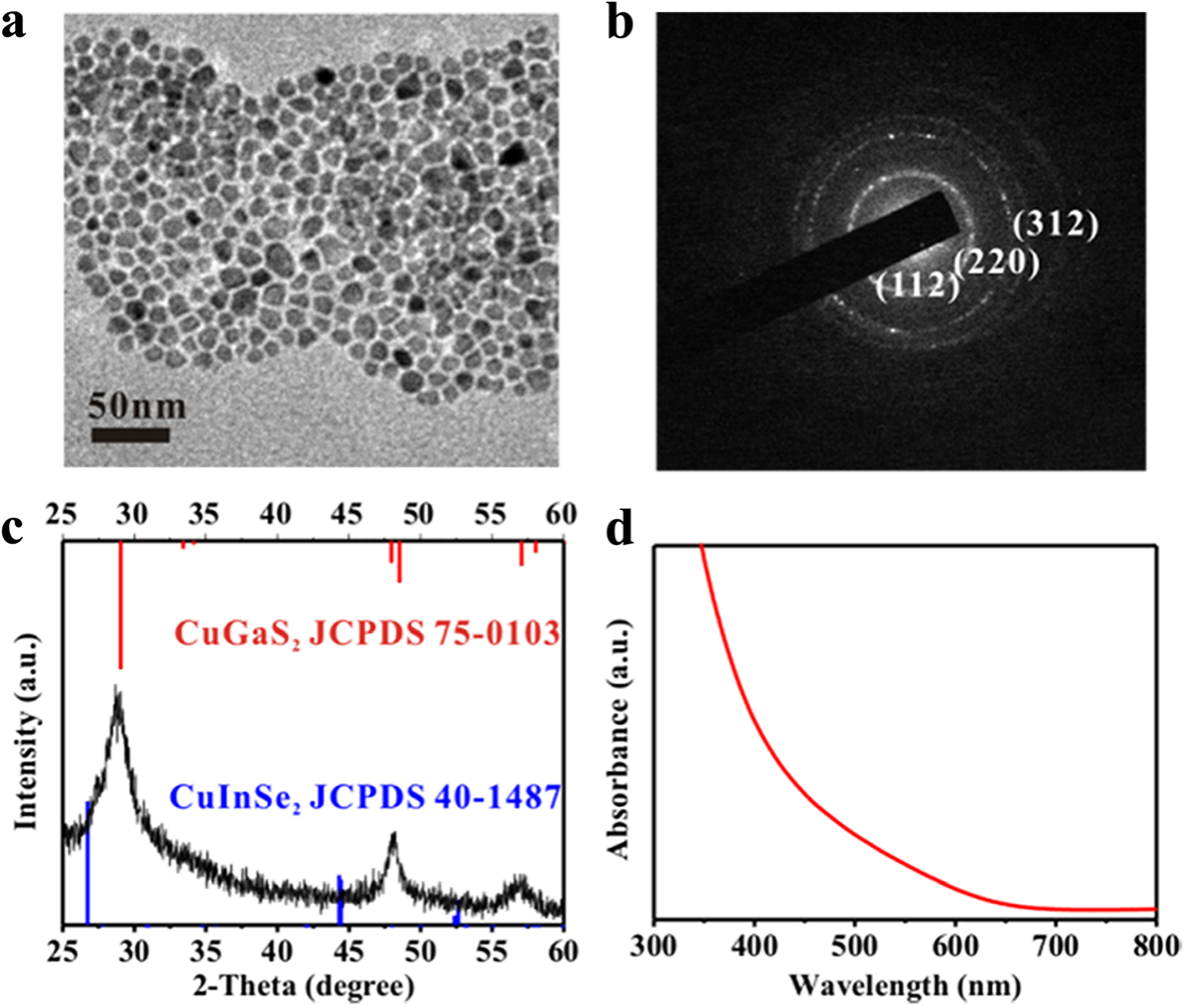
**a** TEM images, **b** SAED pattern, **c** XRD pattern, and **d** UV-Vis absorption spectra of CIGSSe nanocrystals

The size and morphology of as-synthesized CIGSSe nanocrystals were examined by TEM (Fig. 1a). The CIGSSe nanocrystals have an average diameter of 10 nm and a slightly irregular faceted shape. The selected-area electron diffraction pattern (SAED) of a field of nanocrystals (Fig. 1b) shows three main diffraction rings, in agreement with the (112), (220), and (312) of the chalcopyrite phase, respectively. The atomic ratio of Cu:In:Ga:S:Se is nearly 1:0.1:0.9:1.8:0.2, which was determined by energy-dispersive X-ray spectroscopy (EDX) (Additional file 1: Figure S1) of CIGSSe nanocrystals. Figure 1c shows the XRD pattern of the CIGSSe nanocrystals. The diffraction peaks are identified as the tetragonal chalcopyrite structure according to JCPDS 75-0103 and JCPDS 40-1487 data [34]. An intense peak at 29.1° corresponds to the (112) crystal plane. Other prominent peaks correspond to the (204)/(220) and (116)/(312) planes. No other impurities were detected, indicating the phase purity of the product. Figure 1d shows the absorption spectra of CIGSSe nanocrystals dispersed in toluene. The optical bandgap of CIGSSe nanocrystals determined by the intercepts of the tangent of the absorption spectra (see Additional file 1: Figure S2 for the details of bandgap determination) is 2.6 eV. In addition, UPS (Additional file 1: Figure S3) was used to determine the valence band of CIGSSe nanocrystals. The valence band energy (*E*_VB_) of CIGSSe nanocrystals (−5.15 eV) is slightly higher than the *E*_VB_ for CH_3_NH_3_PbI_3_ (−5.43 eV) [2], which favors hole transfer from CH_3_NH_3_PbI_3_ into the inorganic HTM.

**Fig. 2:**
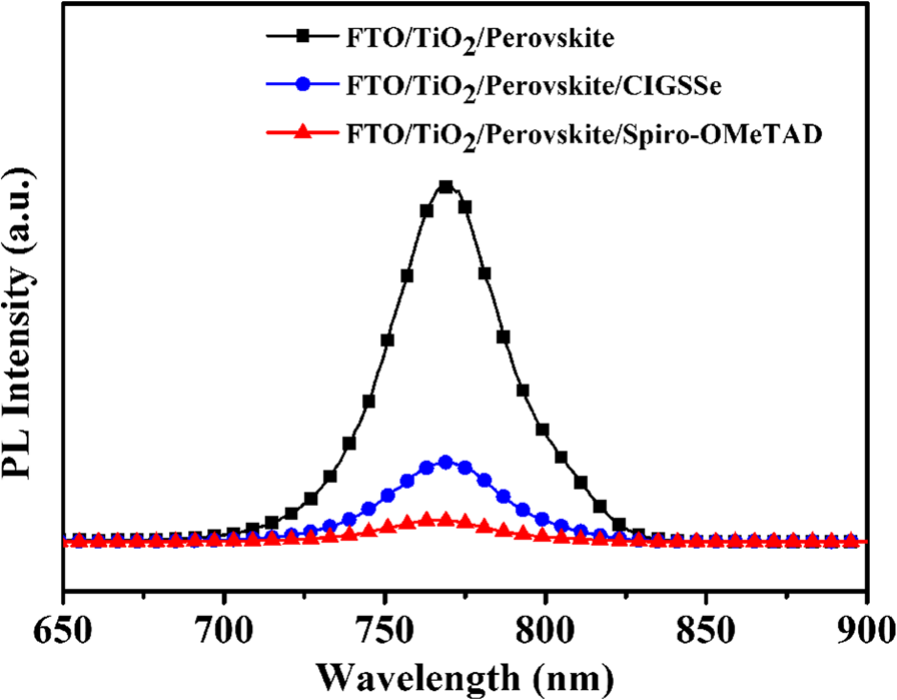
Steady-state photoluminescence (*PL*) spectra for CH_3_NH_3_PbI_3_, CH_3_NH_3_PbI_3_/CIGSSe, and CH_3_NH_3_PbI_3_/ spiro-OMeTAD layers deposited on FTO/bl-TiO_2_/mp-TiO_2_ substrates

Since the CIGSSe nanocrystals are employed as hole-transporting layers in perovskite solar cells, we conducted steady-state photoluminescence (PL) measurements to investigate if CIGSSe film can efficiently extract photo-generated carriers from the perovskite absorber. The carrier extraction efficiency of spiro-OMeTAD was also measured for comparison. As shown in Fig. 2, the steady-state PL intensity of CH_3_NH_3_PbI_3_ is quenched by nearly 78% after CIGSSe deposition. Such a dramatic PL quenching is particularly interesting in solar cell development because it could be caused by the significantly enhanced charge carrier extraction arising from the added CIGSSe layer. Notably, while depositing spiro-OMeTAD on CH_3_NH_3_PbI_3_, the PL intensity decreased further (93%), indicating a more efficient charge transfer process between CH_3_NH_3_PbI_3_ and spiro-OMeTAD layer.

**Fig. 3:**
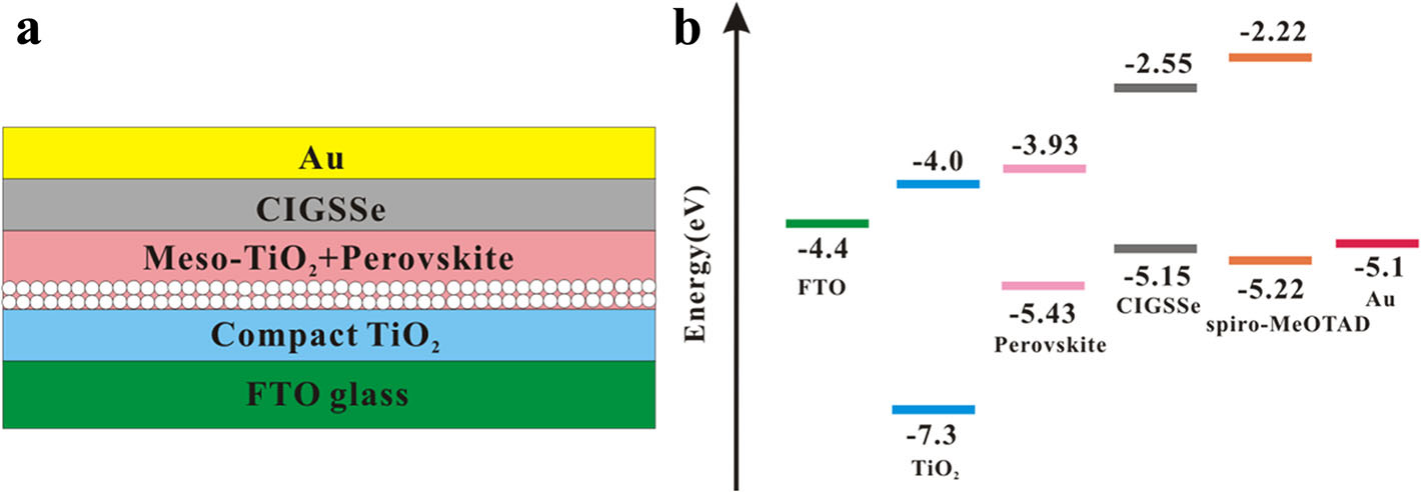
**a** Schematic structure of FTO/bl-TiO_2_/mp-TiO_2_/CH_3_NH_3_PbI_3_/CIGSSe/Au solar cell. **b** Energy-level diagram of various device layers

**Fig. 4:**
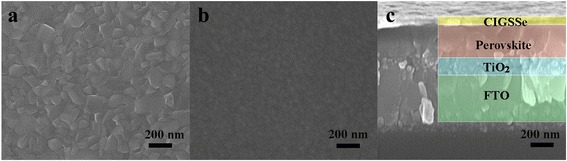
Top view SEM image of **a** CH_3_NH_3_PbI_3_ and **b** CH_3_NH_3_PbI_3_/CIGSSe deposited on the surface of FTO/bl-TiO_2_/mp-TiO_2_. **c** Cross-sectional SEM structure of FTO/bl-TiO_2_/mp-TiO_2_/CH_3_NH_3_PbI_3_/CIGSSe

Perovskite solar cells with the bilayer device structure of FTO/bl-TiO_2_/mp-TiO_2_/CH_3_NH_3_PbI_3_/CIGSSe (or spiro-OMeTAD)/Au (Fig. 3a) were fabricated, and the energy level diagram for the corresponding materials was also depicted in Fig. 3b. Perovskite solar cells utilizing either CIGSSe or spiro-OMeTAD HTMs were constructed by a similar method reported previously in the literature [37]. Briefly, an ∼100-nm compact TiO_2_ blocking layer and an ~200-nm mesoporous TiO_2_ film were deposited on the FTO by spin-coating. The TiO_2_ films were sintered at 550 °C for 30 min. The CH_3_NH_3_PbI_3_ perovskite was deposited on the mesoporous TiO_2_ films by one-step method. For the one-step spin-coating procedure of preparing CH_3_NH_3_PbI_3_ film, a DMF solution containing PbI_2_, CH_3_NH_3_I, and DMSO (1:1:1 mol %) was prepared and then spin-coated on the mesoporous TiO_2_ films to form a transparent adduct film. After heating the film at 65 °C for 1 min and 100 °C for 2 min, it was converted to a dark brown CH_3_NH_3_PbI_3_ film. The crystalline quality of the resulting CH_3_NH_3_PbI_3_ film deposited on the FTO substrate was investigated by XRD spectroscopy (Additional file 1: Figure S4). The diffraction peaks at 14.10°, 20.01°, 23.48°, 24.50°, 28.43°, 31.88°, 40.67°, and 43.20° can be assigned as (110), (112), (211), (202), (220), (310), (224), and (314) planes, respectively, of the CH_3_NH_3_PbI_3_ tetragonal phase, indicating the fully formed perovskite structure from PbI_2_ [35, 38, 39]. The morphology of the CH_3_NH_3_PbI_3_ film was investigated by SEM. As shown in Fig. 4a, the as-formed CH_3_NH_3_PbI_3_ films exhibit full surface coverage and are composed of nanometer-sized grains ranging from tens of nanometers to hundreds of nanometers in size. After deposition of the CIGSSe layer on the perovskite film (Fig. 4b), the film seems homogeneous and the perovskite grains become invisible, suggesting that the perovskite film surface is completely covered by CIGSSe nanocrystals. The cross-sectional SEM image of the FTO/TiO_2_/CH_3_NH_3_PbI_3_/CIGSSe film (Fig. 4c) indicates that the perovskite layer is very compact and has a thickness of ~400 nm, and the CIGSSe HTM layer has a thickness of ~50 nm that covers the perovskite layer uniformly. The full coverage of the perovskite film by CIGSSe and the inhibition of direct contact between the CIGSSe HTM layer and the bottom TiO_2_ layer by compact perovskite layer are expected to be beneficial for prohibiting charge recombination [40]. Finally, an 80-nm Au electrode was thermally evaporated to complete the device.

**Fig. 5:**
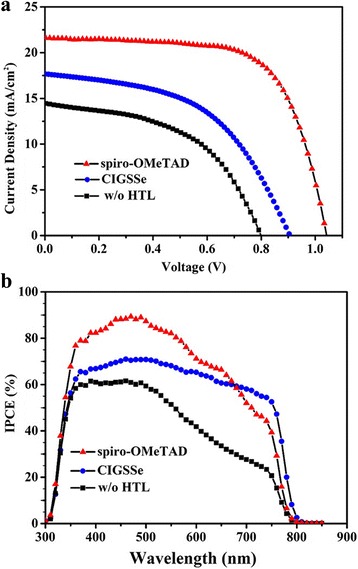
**a** *J*-*V* curves and **b** external quantum efficiency spectra of perovskite solar cells with different HTMs

Figure 5a shows the current density-voltage (*J*-*V*) curves of PSCs employing CIGSSe or spiro-OMeTAD as HTM under AM 1.5 G illumination at 100 mW/cm^2^. The photovoltaic parameters of the PSCs are listed in Table 1. A device without HTM was also fabricated for comparison, which exhibited an open-circuit voltage (*V*_oc_) of 0.80 V, a short-circuit current density (*J*_sc_) of 14.48 mA/cm^2^, and a fill factor (FF) of 50.38%, resulting in a best PCE of 5.85%. A device using CIGSSe as HTM displayed a *V*_oc_ of 0.94 V, a *J*_sc_ of 17.66 mA/cm^2^, and a FF of 54.88%, reaching a best PCE of 9.15%. The overall efficiency enhancement mainly resulted from a significant increase in the *V*_oc_, *J*_sc_, and FF values. Compared with the commonly used spiro-OMeTAD as HTM, the PCE was still low (9.15 vs 15.08%) but higher than that of CuInS_2_ quantum dot-based device [33]. The device performance statistics for devices based on CIGSSe, spiro-OMeTAD, and no HTM were obtained on the basis of 10 independent devices (Additional file 1: Figure S5). The average PCE values follow the same tendency to the highest values discussed above. The external quantum efficiency (EQE) spectra of the devices with various HTMs are plotted in Fig. 5b. Compared with the device without HTM, the EQE spectrum of the device employing CIGSSe or spiro-OMeTAD HTM is significantly improved in the range of 350–800 nm, especially in the longer wavelength region. The increased spectral response should be attributed to the improved charge collection in the presence of HTM. The EQE value for the device using CIGSSe HTM is lower than that for the device using spiro-OMeTAD HTM in the region from 350 to 670 nm. However, the EQE for the device employing CIGSSe HTM is slightly higher than that for the device employing spiro-OMeTAD HTM in the region from 670 to 800 nm, which can be attribute to the smaller bandgap of CIGSSe. Notably, the CIGSSe layer can improve the EQE values of PSCs in the long wavelength region, resulting from photocurrent originating from the CIGSSe layer, which is similar to previous reports about the extension of the photoresponse toward the longer wavelength by CuInS_2_ or Cu_2_ZnSnS_4_ layer [41, 42]. This indicates that CIGSSe can be used to expand the photoresponse of perovskite solar cells. The integrated current density from the EQE spectra for each device is in agreement with the current density obtained from the *J*-*V* curves.

**Table 1:** Photovoltaic parameters of PSCs with different HTMs

HTM	*V*_oc_ (V)	*J*_sc_ (mA/cm^2^)	FF (%)	PCE (%)
CIGSSe	0.94	17.66	54.88	9.15
Spiro-OMeTAD	1.04	21.52	67.19	15.08
Without HTM	0.80	14.48	50.38	5.85

**Fig. 6:**
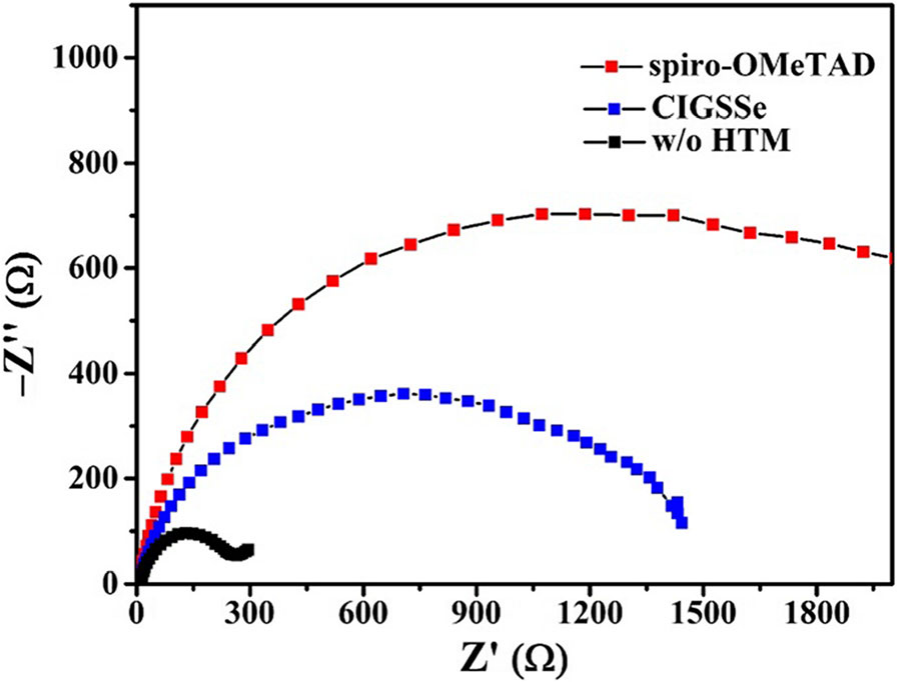
Nyquist plots for perovskite solar cells without HTM and with CIGSSe or spiro-OMeTAD as HTM

We measured impedance spectra of the devices based on different HTMs to describe the recombination processes in PSCs. The recombination resistance (*R*_rec_) can be obtained by fitting the impedance spectra using a simplified equivalent circuit. The main arc at middle frequency is related to a recombination resistance *R*_rec_, in parallel with a chemical capacitance *C*_μ_, related to the recombination between TiO_2_ and HTMs [27, 43, 44]. Impedance spectra for solar cells with CIGSSe and spiro-OMeTAD as HTM and without HTM were recorded over the frequency range of 10 Hz to 1 M Hz under 100 mW/cm^2^ at AM 1.5 G illumination. There is some disorder in the low-frequency data, a common phenomenon in PSCs [45]. As shown in Fig. 6, *R*_rec_ for the device using spiro-OMeTAD or CIGSSe as HTM displays larger value than that for the device without HTM. In addition, *R*_rec_ for the spiro-OMeTAD device shows larger value than that for the CIGSSe device. The larger *R*_rec_ indicates slower recombination in the device, which is an interpretation for the improved efficiency of the CIGSSe and the spiro-OMeTAD device.

**Fig. 7:**
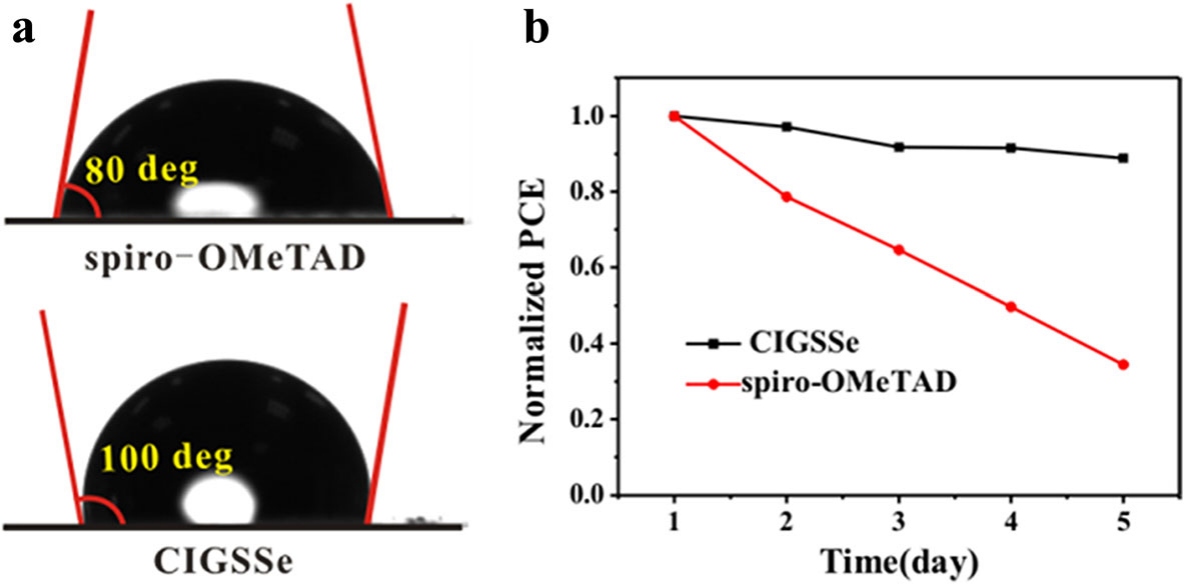
**a** Water contact angles. **b** Normalized PCE of perovskite solar cells employing CIGSSe and spiro-OMeTAD as a function of storage time in air

The long-term stability of perovskite solar cells is very important for practical applications. Although spiro-OMeTAD has been widely used as a HTM in high-performance PSCs, the use of hygroscopic lithium salt doping is unfavorable for device stability [24, 25]. In this respect, the hydrophobic nature of CIGSSe nanocrystals [34, 36] is beneficial for solar cell application. To verify this, the air stability of perovskite solar cells without encapsulation was investigated, as shown in Fig. 7b. When stored in air with a relative humidity of ∼45%, the devices with CIGSSe retained 90% of their original efficiency after 5 days, while the cells with spiro-OMeTAD kept only 36% after 5 days. The difference in device stability resulted from different hydrophobicity of the HTM. The CIGSSe film showed a water contact angle of 100° (Fig. 7a), so that the hydrophobic HTM can efficiently prevent the water penetration into the perovskite layer [46]. By contrast, the spiro-OMeTAD film containing Li-TFSI exhibited the smaller water contact angle of around 80°, indicating an increased affinity of water caused by Li-TFSI. Such hydroscopic ion additive should be avoided in practical applications because of its negative influence on device stability.

## Conclusions

In summary, solution-processed CIGSSe nanocrystals have been successfully applied as a novel Cu-based inorganic HTM for PSCs. The best PCE of the CIGSSe HTM-based device reaches 9.15%, which is the highest PCE for conventional PSCs with Cu-based chalcopyrite semiconductor HTMs reported up to now. In addition, the hydrophobic nature of CIGSSe nanocrystals dramatically enhanced the stability of perovskite solar cells. This work provides a promising candidate of Cu-based inorganic HTM for stable perovskite solar cells.

## Additional file

Additional file 1: The EDX pattern of CIGSSe nanocrystals. Plot of (αhν)^2^ vs photo energy for the CIGSSe nanocrystals. UPS spectra of CIGSSe nanocrystals. XRD pattern of CH_3_NH_3_PbI_3_ film on FTO substrate. Comparison of the performance distributions of 10 individual devices of the cells. (DOCX 398 kb)

## Abbreviations

CIGS: Cu(In_*x*_Ga_1 − *x*_)Se_2_
CIGSSe: CuIn_1 − *x*_Ga_*x*_(S_*y*_Se_1 − *y*_)_2_
*C*_μ_: Chemical capacitance
EDX: Energy-dispersive X-ray spectroscopy
EIS: Electrochemical impedance spectra
EQE: External quantum efficiency
*E*_VB_: Valence band energy
FF: Fill factor
HTM: Hole-transporting material
*J*_sc_: Short-circuit current density
*J*-*V*: Current density-voltage
Li-TFSI: Lithium bis(trifluoromethylsulfonyl)imide
P3HT: Poly(3-hexylthiophene)
PANI: Polyaniline
PCE: Power conversion efficiency
PCPDTBT: Poly[2,1,3-benzothiadiazole-4,7-diyl[4,4-bis(2-ethylhexyl)-4H-cyclopenta[2,1-*b*:3,4-*b*’]dithiophene-2,6-diyl]]
PL: Photoluminescence
PSCs: Perovskite solar cells
PTAA: Polytriarylamine
*R*_rec_: Recombination resistance
SAED: Selected-area electron diffraction pattern
Spiro-OMeTAD: 2,2’,7,7’-tetrakis(*N*,*N*-di-*p*-methoxyphenylamine)-9,9′-spirobifluorene
TEM: Transmission electron microscopy
UPS: Ultraviolet photoelectron spectroscopy
*V*_oc_: Open-circuit voltage
XRD: X-ray diffraction

## References

[1] Kojima A, Teshima K, Shirai Y, Miyasaka T (2009). Organometal halide perovskites as visible-light sensitizers for photovoltaic cells. J Am Chem Soc.

[2] Kim HS, Lee CR, Im JH, Lee KB, Moehl T, Marchioro A, Moon SJ, Humphry-Baker R, Yum JH, Moser JE, Grätzel M, Park NG (2012). Lead iodide perovskite sensitized all-solid-state submicron thin film mesoscopic solar cell with efficiency exceeding 9%. Sci Rep.

[3] Yin W-J, Yang J-H, Kang J, Yan Y, Wei S-H (2015). Halide perovskite materials for solar cells: a theoretical review. J Mater Chem A.

[4] Wehrenfennig C, Eperon GE, Johnston MB, Snaith HJ, Herz LM (2014). High charge carrier mobilities and lifetimes in organolead trihalide perovskites. Adv Mater.

[5] Stranks SD, Eperon GE, Grancini G, Menelaou C, Alcocer MJ, Leijtens T, Herz LM, Petrozza A, Snaith HJ (2013). Electron-hole diffusion lengths exceeding 1 micrometer in an organometal trihalide perovskite absorber. Science.

[6] Xing G, Mathews N, Sun S, Lim SS, Lam YM, Grätzel M, Mhaisalkar S, Sum TC (2013). Long-range balanced electron- and hole-transport lengths in organic-inorganic CH_3_NH_3_PbI_3_. Science.

[7] Grätzel M (2014). The light and shade of perovskite solar cells. Nat Mater.

[8] Habibi M, Zabihi F, Ahmadian-Yazdi MR, Eslamian M (2016). Progress in emerging solution-processed thin film solar cells—part II: perovskite solar cells. Renew Sust Energy Rev.

[9] Zuo C, Bolink HJ, Han H, Huang J, Cahen D, Ding L (2016). Advances in perovskite solar cells. Adv Sci.

[10] Stranks SD, Nayak PK, Zhang W, Stergiopoulos T, Snaith HJ (2015). Formation of thin films of organic-inorganic perovskites for high-efficiency solar cells. Angew Chem Int Ed.

[11] Ahmadian-Yazdi MR, Zabihi F, Habibi M, Eslamian M (2016). Effects of process parameters on the characteristics of mixed-halide perovskite solar cells fabricated by one-step and two-step sequential coating. Nanoscale Res Lett.

[12] Zabihi F, Ahmadian-Yazdi M-R, Eslamian M (2016). Fundamental study on the fabrication of inverted planar perovskite solar cells using two-step sequential substrate vibration-assisted spray coating (2S-SVASC). Nanoscale Res Lett.

[13] Im JH, Lee CR, Lee JW, Park SW, Park NG (2011). 6.5% efficient perovskite quantum-dot-sensitized solar cell. Nanoscale.

[14] Lee MM, Teuscher J, Miyasaka T, Murakami TN, Snaith HJ (2012). Efficient hybrid solar cells based on meso-superstructured organometal halide perovskites. Science.

[15] Liu M, Johnston MB, Snaith HJ (2013). Efficient planar heterojunction perovskite solar cells by vapour deposition. Nature.

[16] Zhou H, Chen Q, Li G, Luo S, Song TB, Duan HS, Hong Z, You J, Liu Y, Yang Y (2014). Interface engineering of highly efficient perovskite solar cells. Science.

[17] Chen W, Wu Y, Yue Y, Liu J, Zhang W, Yang X, Chen H, Bi E, Ashraful I, Grätzel M (2015). Efficient and stable large-area perovskite solar cells with inorganic charge extraction layers. Science.

[18] Nie W, Tsai H, Asadpour R, Blancon J-C, Neukirch AJ, Gupta G, Crochet JJ, Chhowalla M, Tretiak S, Alam MA (2015). High-efficiency solution-processed perovskite solar cells with millimeter-scale grains. Science.

[19] Yang WS, Noh JH, Jeon NJ, Kim YC, Ryu S, Seo J, Seok SI (2015). High-performance photovoltaic perovskite layers fabricated through intramolecular exchange. Science.

[20] Heo JH, Im SH, Noh JH, Mandal TN, Lim C-S, Chang JA, Lee YH, H-j K, Sarkar A, Nazeeruddin MK (2013). Efficient inorganic-organic hybrid heterojunction solar cells containing perovskite compound and polymeric hole conductors. Nat Photon.

[21] Di Giacomo F, Razza S, Matteocci F, D’Epifanio A, Licoccia S, Brown TM, Di Carlo A (2014). High efficiency CH_3_NH_3_PbI_(3-x)_Cl x perovskite solar cells with poly (3-hexylthiophene) hole transport layer. J Power Sources.

[22] Zhang Y, Liu W, Tan F, Gu Y (2015). The essential role of the poly (3-hexylthiophene) hole transport layer in perovskite solar cells. J Power Sources.

[23] Xiao Y, Han G, Chang Y, Zhou H, Li M, Li Y (2014). An all-solid-state perovskite-sensitized solar cell based on the dual function polyaniline as the sensitizer and p-type hole-transporting material. J Power Sources.

[24] Noh JH, Im SH, Heo JH, Mandal TN, Seok SI (2013). Chemical management for colorful, efficient, and stable inorganic–organic hybrid nanostructured solar cells. Nano Lett.

[25] Niu G, Li W, Meng F, Wang L, Dong H, Qiu Y (2014). Study on the stability of CH_3_NH_3_PbI_3_ films and the effect of post-modification by aluminum oxide in all-solid-state hybrid solar cells. J Mater Chem A.

[26] Qin P, Tanaka S, Ito S, Tetreault N, Manabe K, Nishino H, Nazeeruddin MK, Grätzel M (2014). Inorganic hole conductor-based lead halide perovskite solar cells with 12.4% conversion efficiency. Nat Commun.

[27] Christians JA, Fung RC, Kamat PV (2013). An inorganic hole conductor for organo-lead halide perovskite solar cells. Improved hole conductivity with copper iodide. J Am Chem Soc.

[28] Chen WY, Deng LL, Dai SM, Wang X, Tian CB, Zhan XX, Xie SY, Huang RB, Zheng LS (2015). Low-cost solution-processed copper iodide as an alternative to PEDOT:PSS hole transport layer for efficient and stable inverted planar heterojunction perovskite solar cells. J Mater Chem A.

[29] Li Y, Zhu J, Huang Y, Wei J, Liu F, Shao Z, Hu L, Chen S, Yang S, Tang J, Yao J, Dai S (2015). Efficient inorganic solid solar cells composed of perovskite and PbS quantum dots. Nanoscale.

[30] Kim JH, Liang PW, Williams ST, Cho N, Chueh CC, Glaz MS, Ginger DS, Jen AK (2015). High-performance and environmentally stable planar heterojunction perovskite solar cells based on a solution-processed copper-doped nickel oxide hole-transporting layer. Adv Mater.

[31] Chopra K, Paulson P, Dutta V (2004). Thin-film solar cells: an overview. Prog Photovolt Res Appl.

[32] Romeo A, Terheggen M, Abou‐Ras D, Bätzner D, Haug FJ, Kälin M, Rudmann D, Tiwari A (2004). Development of thin-film Cu (In, Ga)Se_2_ and CdTe solar cells. Prog Photovolt Res Appl.

[33] Lv M, Zhu J, Huang Y, Li Y, Shao Z, Xu Y, Dai S (2015). Colloidal CuInS_2_ quantum dots as inorganic hole-transporting material in perovskite solar cells. ACS Appl Mater Interfaces.

[34] Chang S-H, Chiang M-Y, Chiang C-C, Yuan F-W, Chen C-Y, Chiu B-C, Kao T-L, Lai C-H, Tuan H-Y (2011). Facile colloidal synthesis of quinary CuIn_1-x_Ga_x_(S_y_Se_1-y_)_2_ (CIGSSe) nanocrystal inks with tunable band gaps for use in low-cost photovoltaics. Energy Environ Sci.

[35] Jeon NJ, Noh JH, Kim YC, Yang WS, Ryu S, Seok SI (2014). Solvent engineering for high-performance inorganic-organic hybrid perovskite solar cells. Nat Mater.

[36] Panthani MG, Akhavan V, Goodfellow B, Schmidtke JP, Dunn L, Dodabalapur A, Barbara PF, Korgel BA (2008). Synthesis of CuInS_2_, CuInSe_2_, and Cu(In_x_Ga_1-x_)Se_2_ (CIGS) nanocrystal “inks” for printable photovoltaics. J Am Chem Soc.

[37] Ahn N, Son DY, Jang IH, Kang SM, Choi M, Park NG (2015). Highly reproducible perovskite solar cells with average efficiency of 18.3% and best efficiency of 19.7% fabricated via Lewis base adduct of lead(II) iodide. J Am Chem Soc.

[38] Liang K, Mitzi DB, Prikas MT (1998). Synthesis and characterization of organic-inorganic perovskite thin films prepared using a versatile two-step dipping technique. Chem Mater.

[39] Burschka J, Pellet N, Moon S-J, Humphry-Baker R, Gao P, Nazeeruddin MK, Grätzel M (2013). Sequential deposition as a route to high-performance perovskite-sensitized solar cells. Nature.

[40] Xiao M, Huang F, Huang W, Dkhissi Y, Zhu Y, Etheridge J, Gray-Weale A, Bach U, Cheng Y-B, Spiccia L (2014). A fast deposition-crystallization procedure for highly efficient lead iodide perovskite thin-film solar cells. Angew Chem Int Ed.

[41] Chen C, Li C, Li F, Wu F, Tan F, Zhai Y, Zhang W (2014). Efficient perovskite solar cells based on low-temperature solution-processed (CH_3_NH_3_)PbI_3_ perovskite/CuInS_2_ planar heterojunctions. Nanoscale Res Lett.

[42] Wu Q, Xue C, Li Y, Zhou P, Liu W, Zhu J, Dai S, Zhu C, Yang S (2015). Kesterite Cu_2_ZnSnS_4_ as a low-cost inorganic hole-transporting material for high-efficiency perovskite solar cells. ACS Appl Mater Interfaces.

[43] Kim HS, Mora-Sero I, Gonzalez-Pedro V, Fabregat-Santiago F, Juarez-Perez EJ, Park NG, Bisquert J (2013). Mechanism of carrier accumulation in perovskite thin-absorber solar cells. Nat Commun.

[44] Kim HS, Lee JW, Yantara N, Boix PP, Kulkarni SA, Mhaisalkar S, Grätzel M, Park NG (2013). High efficiency solid-state sensitized solar cell-based on submicrometer rutile TiO_2_ nanorod and CH_3_NH_3_PbI_3_ perovskite sensitizer. Nano Lett.

[45] Liu D, Yang J, Kelly TL (2014). Compact layer free perovskite solar cells with 13.5% efficiency. J Am Chem Soc.

[46] Kwon YS, Lim J, Yun H-J, Kim Y-H, Park T (2014). A diketopyrrolopyrrole-containing hole transporting conjugated polymer for use in efficient stable organic-inorganic hybrid solar cells based on a perovskite. Energy Environ Sci.

