# Supplementary material for: Solution-Processed Cu(In, Ga)(S, Se)2 Nanocrystal as Inorganic Hole-Transporting Material for Efficient and Stable Perovskite Solar Cells
Source: Nanoscale Res Lett. 2017 Feb 28;12:159. doi: 10.1186/s11671-017-1933-z (PMC5331025; doi:10.1186/s11671-017-1933-z)
Supplement: Additional file 1: — The EDX pattern of CIGSSe nanocrystals. Plot of (αhν)2 vs photo energy for the CIGSSe nanocrystals. UPS spectra of CIGSSe nanocrystals. XRD pattern of CH3NH3PbI3 film on FTO substrate. Comparison of the performance distributions of 10 individual devices of the cells. (DOCX 398 kb) [file 11671_2017_1933_MOESM1_ESM.docx]

**Supporting Information**

**Solution-Processed Cu(In, Ga)(S, Se)_2_ Nanocrystal as Inorganic Hole-Transporting Material for Efficient and Stable Perovskite Solar Cells**

Lu Xu^1^, Lin-Long Deng^2^*, Jing Cao^1^, Xin Wang^2^, Wei-Yi Chen^2^, and Zhiyuan Jiang^1^*

^1^State Key Laboratory of Physical Chemistry of Solid Surfaces, Department of Chemistry, College of Chemistry and Chemical Engineering, Xiamen University, Xiamen, 361005, P.R. China.

^2^Pen-Tung Sah Institute of Micro-Nano Science and Technology, Xiamen University, Xiamen 361005, P. R. China.

*Correspondence: denglinlong@xmu.edu.cn; zyjiang@xmu.edu.cn

**
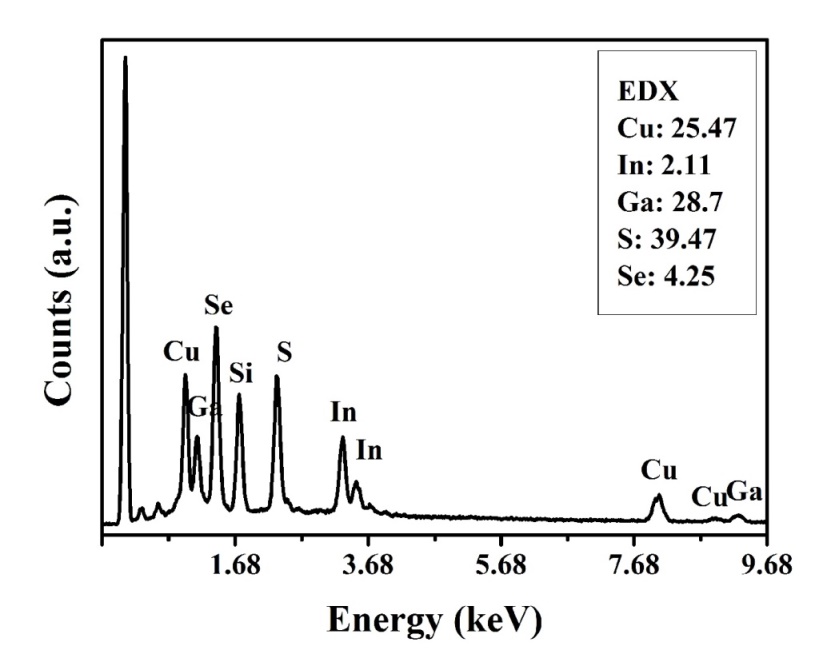
**

**Figure S1.** The EDX pattern of CIGSSe nanocrystals.

**Determination of optical band gap from UV-Vis spectra:**

The direct allowed optical band gap E_g_ can be determined with the relation:[[1](#_ENREF_1)]

$$\alpha h\upsilon=D{(h\upsilon-E_{g})}^{n}$$

where D is a constant, hν is the photon energy, and E_g_ is the optical band gap.

For a direct transition, n=1/2. By extrapolating the linear part of the plot to (αhν)^2^ = 0, value of the direct band gap E_g_ was determined. The experimental value of (αhν)^2^ against hν is plotted in Figure S2.


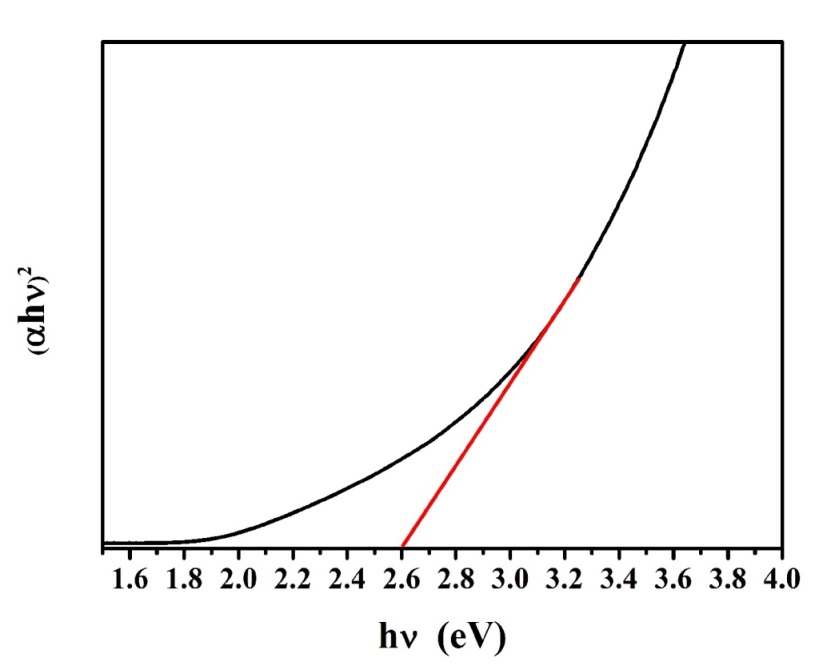


**Figure S2.** Plot of (αhν)^2^ vs photo energy for the CIGSSe nanocrystals.


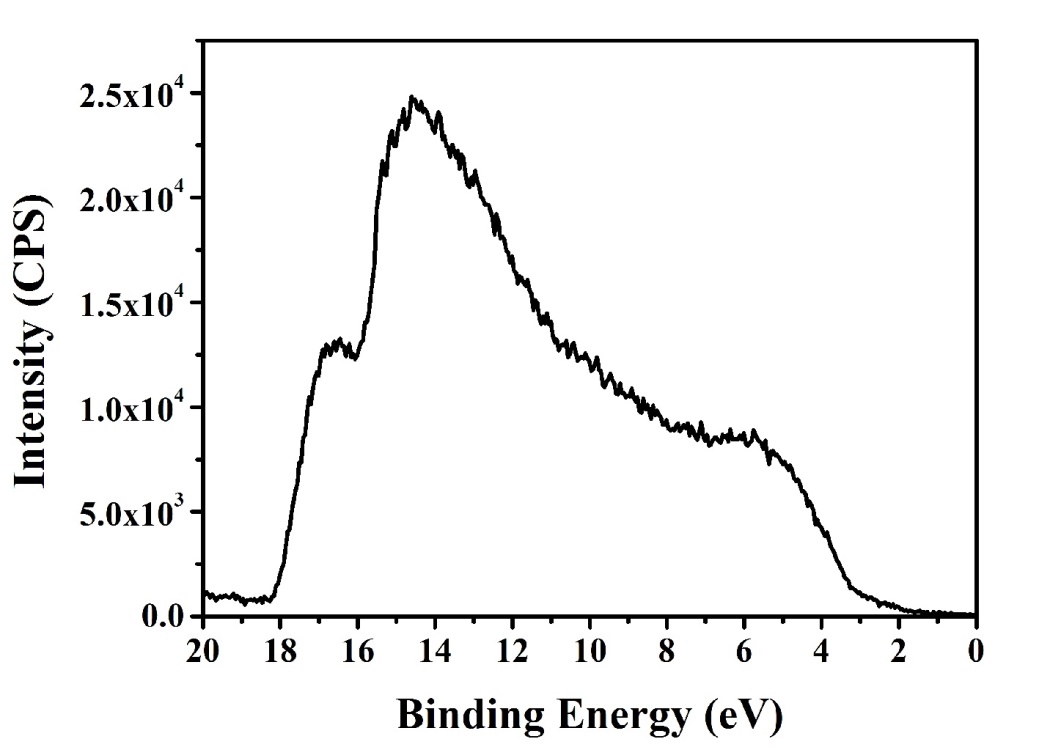


**Figure S3.** UPS spectra of CIGSSe nanocrystals.


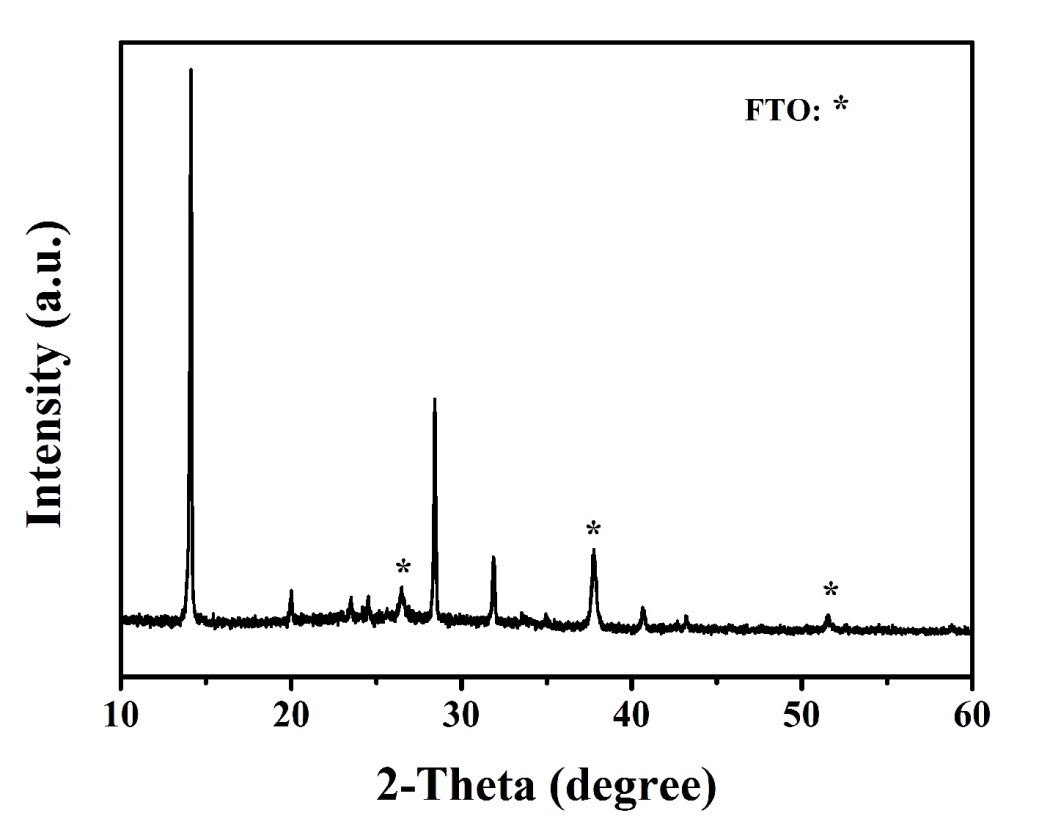


**Figure S4.** XRD pattern of CH_3_NH_3_PbI_3_ film on FTO substrate.


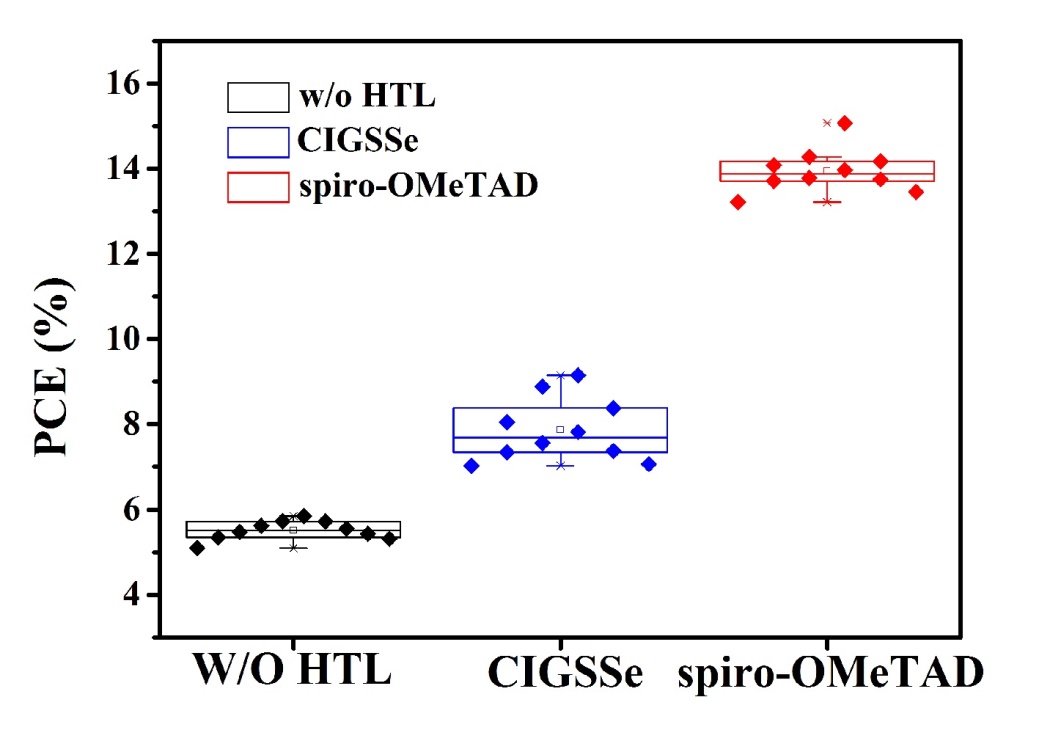


**Figure S5.** Comparison of the performance distributions of 10 individual devices of the cells.

References:

[1] Tan S,Chen B,Sun X,Fan W,Kwok HS,Zhang X,Chua S (2005) Blueshift of optical band gap in ZnO thin films grown by metal-organic chemical-vapor deposition. J. Appl. Phys. 98: 13505-13505.
